# Computational Study of PCSK9-EGFA Complex with Effective Polarizable Bond Force Field

**DOI:** 10.3389/fmolb.2017.00101

**Published:** 2018-01-15

**Authors:** Jian Chen, Lili Duan, Changge Ji, John Z. H. Zhang

**Affiliations:** ^1^Shanghai Engineering Research Center for Molecular Therapeutics and New Drug Development, School of Chemistry and Molecular Engineering, East China Normal University, Shanghai, China; ^2^School of Physics and Electronics, Shandong Normal University, Jinan, China; ^3^NYU-ECNU Center for Computational Chemistry at NYU Shanghai, Shanghai, China

**Keywords:** low density lipoprotein receptor (LDLR), proprotein convertase subtilisin/kexin-type 9 (PCSK9), PBSA, effective polarizable bond (EPB)

## Abstract

Inhibiting of Proprotein Convertase Subtilisin/Kexin-type 9 (PCSK9) and Low Density Lipoprotein Receptor (LDLR) binding is an effective way for reducing Low Density Lipoprotein cholesterol (LDL-C). Understanding the interaction between PCSK9 and LDLR is useful for PCSK9 inhibitor design. In this work, MD simulations with the standard (non-polarizable) AMBER force field and effective polarizable bond (EPB) force field were performed for wild type and four mutants of PCSK9 and EGFA (Epidermal Growth Factor-like repeat A) domain of LDLR complexes. These four mutants are gain-of-function mutants. The analysis of hydrogen bond dynamics and the relative binding free energy indicates that EPB is more reliable in simulating protein dynamics and predicting relative binding affinity. Structures sampled from MD simulations with the standard AMBER force field deviate too far away from crystal structures. Many important interaction components between of PCSK9 and EGFA no longer exist in the simulation with the Amber force field. For comparison, simulation using EPB force field gives more stable structures as shown by hydrogen bond analysis and produced relative binding free energies that are consistent with experimental results. Our study suggests that inclusion of polarization effects in MD simulation is important for studying the protein-protein interaction.

## Introduction

Cholesterol is an important substance of life. It is carried by lipoproteins to where it needs to go and functions in cell membrane, cell signaling, and nerve conduction. However, it is known that the low density lipoprotein (LDL) cholesterol is the “bad” cholesterol. When the concentrations of plasma LDL cholesterol (LDL-C) are too high *in vivo*, they gather on the walls of the blood vessels and may cause blockages. Higher LDL levels suggest a greater risk of cardiovascular disease (CVD), such as hyperlipemia and atherosclerosis.

The low-density lipoprotein receptor (LDLR) is a transmembrane protein, it binds to circulating LDL, and the LDLR/LDL complex is internalized by clathrin-mediated endocytosis. At low pH in the endosomes, the LDLR/LDL complex dissociates allowing receptor recycling and lysosomal degradation of LDL. LDLR is the primary worker for removal of cholesterol from the circulation (Brown et al., 1997; Lagor and Millar, 2010). The LDLR is a 839-amino-acids protein and contains 5 domains: (1) LDLR type A repeat domains, (2) epidermal growth factor (EGF) receptor homology domain containing the β-propeller subdomain, (3) O-linked glycosylation domain, (4) transmembrane domain, and (5) cytoplasmic domain containing NPXY sequence (Kwon et al., 2008). Each domain plays different roles in circulating LDL.

Proprotein convertase subtilisin-like kexin type 9 (PCSK9) is a secreted protease that binds to and promotes degradation of the LDLR protein. It contains an N-terminal signal sequence, a pro-domain, a catalytic domain, and a cysteine-rich C-terminal domain. When PCSK9 binds to the LDLR, the PCSK9/LDLR complex could not dissociate and the receptor is destroyed along with the LDL particle. LDLR can no longer remove LDL-C from the blood (Cunningham et al., 2007). If PCSK9 is blocked, more LDLRs would accumulate on the surface of the liver and would remove more LDL-C from the blood (Costet et al., 2008). Gain-of-function mutations in PCSK9 that enhance its interaction with the LDLR result in markedly higher LDL-C levels in humans. Therefore, blocking PCSK9 can lower blood cholesterol levels.

Statins have been discovered to reduce cardiovascular disease and mortality in those who are at high risk. But statins may be less effective in reducing LDL cholesterol in people with FH (Familial Hypercholesterolemia). Drugs that target PCSK9 can lead to lowered circulating cholesterol. Therefore, PSCK9 inhibitor is one of the most promising emerging treatment options. The epidermal growth factor-like repeat A (EGFA) of the LDLR is sufficient for PCSK9 binding and the PCSK9 C-terminal domain is not involved in LDLR binding (Zhang et al., 2007; Holla et al., 2011). Bottomley et al. reported the neutral pH x-ray crystal structure of WT C-terminal removed PCSK9ΔC bound to EGFA and the structures of gain-of-function mutant forms of these proteins associated with FH (Familial Hypercholesterolemia), including EGFA bound to PCSK9ΔCD374A, PCSK9ΔCD374Y, and PCSK9ΔCD374H, and of WT PCSK9 bound to EGFAH306Y (Bottomley et al., 2008).

In the current study, we present MD simulation and binding affinity analysis of the complexes PCSK9ΔC-EGFA, PCSK9ΔCD374A-EGFA, PCSK9ΔCD374Y-EGFA, PCSK9ΔCD374H-EGFA, and PCSK9ΔC-EGFAH306Y to investigate the details of the interaction between PCSK9 and EGFA. We try to get the reason of why these mutations enhance the interaction and to provide useful information for finding of peptides mimics the EGFA domain of the LDLR that binds to PCSK9 to inhibit PCSK9.

## Methods

### Simulations with the standard amber force field

**Figure 1:**
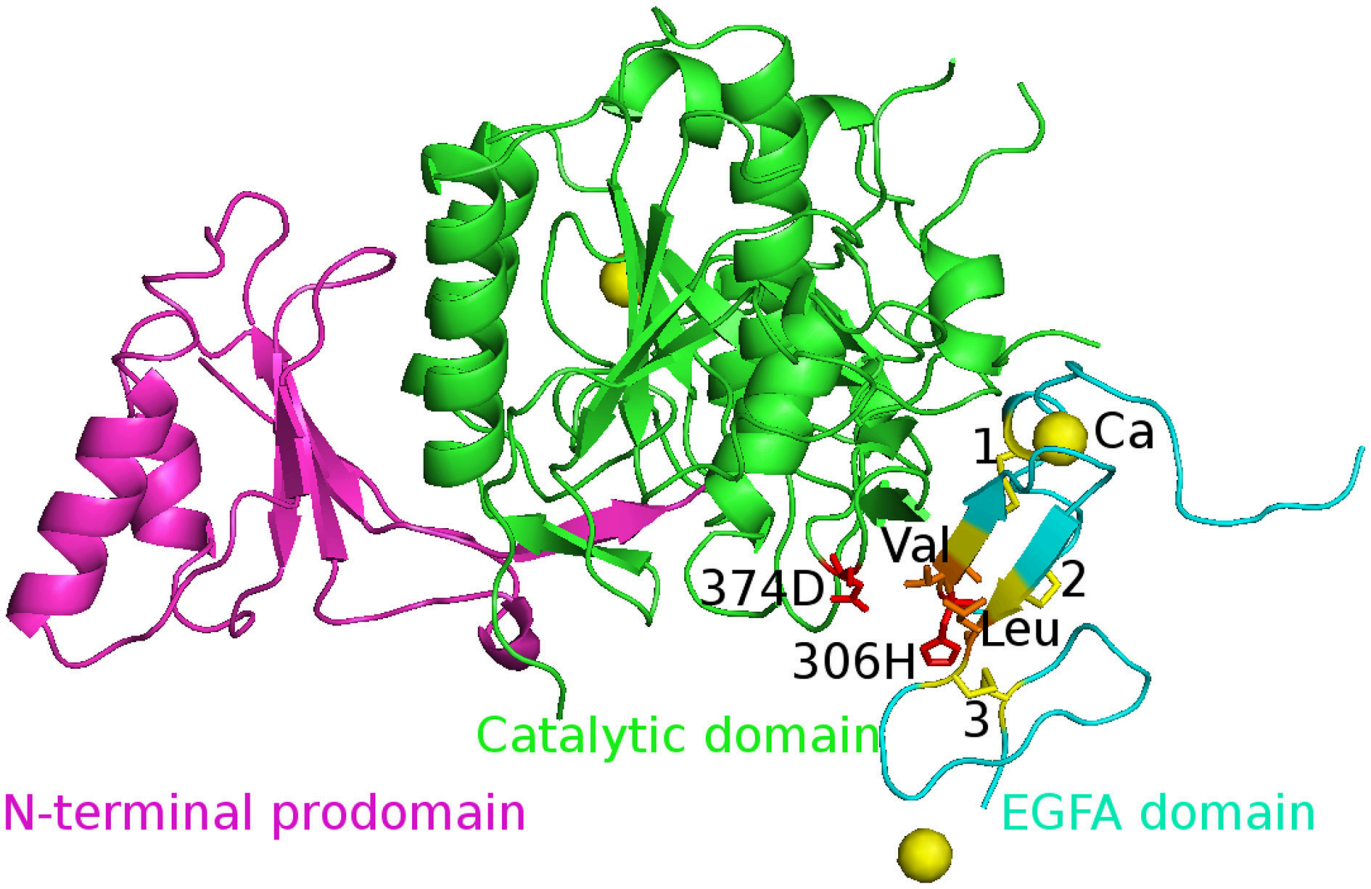
The initial structure of the complex WT PCSK9ΔC-EGFA. PCSK9ΔC has two domains: N-terminal prodomain (in magenta) and Catalytic domain (in green). There are three disulfide bonds (yellow bond 1, 2, 3) in EGFA domain (in cyan). The residues 374D in PCSK9 catalytic domain and 306H in EGFA domain are highlighted in red stick and two important residues Val and Leu are in orange. The Ca^2+^ ions are shown in yellow ball. The four mutants' structures are similar to this figure by changing the red residues in the mutation site.

The initial structures of the five complexes were taken from the Protein Data Bank (PDB) and PDB code is 2W2M (WT), 2W2Q (D374H), 2W2N (H306Y), 2W2O (D374Y), and 2W2P (D374A) respectively. The chain B was deleted and all crystal water molecules and Ca^2+^ ions were kept in the simulations. The missing atoms were added by Pymol. The structure of WT PCSK9ΔC-EGFA complex is shown in Figure 1. Three disulfide bonds (yellow bond 1, 2, 3 as shown in Figure 1) were built in the model. The residues 374D in PCSK9 catalytic domain and 306H in EGFA domain were highlighted in red stick.

MD simulation was carried out using the AMBER 12 package with AMBER14SB force field at room temperature. The PCSK9-EGFA complexes as well as the mutated complexes were constructed as described above. The Leap module was used to add hydrogen atoms. The protein systems were solvated in octahedron-like TIP3P (Mahoney and Jorgensen, 2000) water box and were neutralized by adding counter-ions. The distance from the surfaces of the box to the closest atoms of the solutes was set to 10 Å. Periodic boundary conditions and the Particle Mesh Ewald (PME) (Darden et al., 1993; John Wiley Sons Ltd., 2002) methods were used to treat long-range electrostatic effects and the van der Waals interactions were truncated at 10 Å. All systems were relaxed in a two-step equilibration procedure. First, only the solvent molecules were optimized by holding the solute fixed with an external force for 5,000 steps of steepest descent minimization, followed by 45,000 steps of conjugate gradient minimization. Then, the whole systems were optimized without constraint until the convergence reached. After this dual-step equilibration, the systems were heated from 0 K up to 300 K in 200 ps with harmonic constraints on all solute atoms, then NPT MD simulations were performed for 30 ns using AMBER force field without any restraints on solute atoms at 300 K and 1 MPa with a time step of 2 fs. Langevin dynamics were applied to regulate the temperature with a collision frequency of 1.0 ps^−1^. The SHAKE algorithm (Andersen, 1983) was applied to restrain all bonds involving hydrogen atoms.

### Simulation with the effective polarizable bond (EPB) force field

The electrostatic interaction plays critical role in stabilizing protein complexes (Perutz, 1978; Honig and Nicholls, 1995; Halgren, 2001). The strength of electrostatic interaction depends on the polarization state of the polarizable dipoles. Traditional force fields use fixed partial charge which is weak in describing the environment dependent character of polar interactions. Many efforts have been made to incorporate polarization effects in molecular simulation in the past decades (Kaminski et al., 2002; Yu and van Gunsteren, 2005; Cieplak et al., 2009; Ji and Zhang, 2009; Duan et al., 2010, 2012; Tong et al., 2010; Wang et al., 2011; Xiang et al., 2011; Ji et al., 2012). Effective polarizable bond (EPB) model is a recent development along this direction (Ji et al., 2012; Xiao et al., 2013). Partial charges were allowed to fluctuate during MD simulation along a polarizable bond according its electrostatic environment. In this partial polarizable approach, all polar groups of amino acids were treated as polarizable bond in MD simulation, and the relevant polarizable parameters were determined by fitting to quantum calculated electrostatic properties of these polar groups. A detailed description of this method could be found in the references (Ji et al., 2012; Xiao et al., 2013). A brief description of effective polarizable bond (EPB) model is given below.

Consider transferring a polar group such as SH from gas phase into liquid phase, the energy of the this polar system can be written as

(1) $$\begin{array}{l} E=E_{self}+E_{ele}\\ \;=\left[k{\left(\mu_{liquid}-\mu_{gas}\right)}^{2}\right]+\left[q_{S}\Phi_{S}+q_{H}\Phi_{H}\right] \end{array}$$

where *q*_*S*_ and *q*_*H*_ are, respectively, the ESP (electrostatic potential) charges of S and H atoms of the –SH group. The Φ_*S*_ and Φ_*H*_ are electrostatic potentials at, respectively, S and H atoms. In the present approach, the polarization can be treated as charge transfer between atoms of a polar group. If the amount of charge transfer from atom S to atom H is Δq, as in the –SH group, the final partial charges of the atoms are given by

(2) $$\begin{array}{r} q_{S}=q_{S}^{gas}+\Delta q \end{array}$$

(3) $$\begin{array}{r} q_{H}=q_{H}^{gas}-\Delta q \end{array}$$

where $q_{S}^{gas}$ and $q_{H}^{gas}$ are, respectively, the atomic charges of S and H atoms in gas phase (or reference charges). Thus, the change of dipole moment of the –SH group due to polarization (from gas phase to solvent) is given by

(4) $$\begin{array}{r} \Delta \mu =\mu_{liquid}-\mu_{gas}=\Delta q\cdot d_{SH} \end{array}$$

where *d*_*SH*_ is is the bond length of the S-H bond. Then Equation (1) can be rewritten as,

(5) $$\begin{array}{r} E=E_{0}+k{\left(\Delta q\cdot d_{SH}\right)}^{2}+\left(q_{S}^{gas}+\Delta q\right)\Phi_{S}+\left(q_{H}^{gas}-\Delta q\right)\Phi_{H} \end{array}$$

Minimization of Equation (5) leads to

(6) $$\begin{array}{r} \Delta q=\frac{\left(\Phi_{H}-\Phi_{S}\right)}{2{d_{SH}}^{2}k} \end{array}$$

The specific polarization parameters are predetermined from large scale quantum chemistry calculation of model molecules under different electrostatic environments.

In this paper, several systems were studied using the EPB model to investigate polarization effects in proteins. It was found that protein structure and dynamics were better described in the simulation using the fluctuating charge than using traditional AMBER99SB (Hornak et al., 2006) charge. In this work, after a two-step equilibration procedure and a system's heated-up, the 10 ns MD simulations were performed using the force field of AMBER99SB mixed with the EPB model. Electrostatic potential on each atom was accumulated in the simulation for calculation of the fluctuating charge. The modified version of AMBER12 package was used as a computational tool for simulation with EPB model.

### MM/PBSA

MM/PBSA is widely used in estimating relative binding energy of protein-ligand and protein-protein complexes (Genheden and Ryde, 2015; Duan et al., 2016a,b, 2017a,b). In the MM/PBSA calculation, the average total free energy of the system, G, is evaluated as

(7) $$\begin{array}{r} G=E_{es}+G_{PB}+E_{vdW}+G_{np}-TS_{solute} \end{array}$$

where *G* is decomposed into contributions from electrostatic (*E*_*es*_), van der Waals (*E*_*vdW*_), polar solvation (*G*_*PB*_), non-polar solvation (*G*_*np*_), and entropy (*TS*_*solute*_) term. The binding free energy of a non-covalent association, Δ*G*_*bind*_, can be computed as

(8) $$\begin{array}{l} \Delta G_{bind}=G_{complex}-G_{receptor}-G_{ligand}\\ \;=\Delta G_{PBSA}-T\Delta S_{solute}\\ \;=\Delta E_{es}+\Delta G_{PB}+\Delta E_{vdW}+\Delta G_{np}-T\Delta S_{solute} \end{array}$$

For the MM/PBSA calculations using standard amber force field, the five systems were carried out for 30 ns MD simulations and reached their equilibrium states. Fifty snapshots were extracted from the last 5 ns MD trajectory to calculate Δ*G*_PBSA_ energy of the five systems using the MM/PBSA programs. It is well-known that the MM-PBSA method is very slow to obtain the converged result (Deng and Cieplack, 2009). To check that the systems are fully equilibrated and the data to calculate thermodynamic properties are reliable, We did the analysis of the binding free energy vs. simulation time from the current trajectories of these five systems and the plots are shown in Figure S1. We only examine the convergence in the G_PBSA_ because the solute entropy calculation require high computational cost. Although there are small drifts on the plots, due to the short simulation time, the binding free energy still at least qualitatively can be trust. Normal mode analysis was used to calculate entropy contribution for binding. Since normal mode analysis is extremely time consuming for large systems, only 5 snapshots picked from 50 snapshots were used in estimating *T*Δ*S*_*solute*_ energy.

Similar to the above calculations, binding free energy analysis was also performed on the five complexes using EPB trajectory. Although the absolute values of the calculated binding free energy may not be accurate, the relative values can still provide us some useful information. All the relative energy components compared to the wild type complex were listed in Table 1. Each relative binding energy term is expressed as follows:

(9) $$\begin{array}{r} \Delta \Delta G=\Delta G_{bind}\left(mutant\right)-\Delta G_{bind}\left(WT\right) \end{array}$$

**Table 1:** Binding free energies between PCSK9ΔC and EGFA using standard AMBER charge and EPB.

**Systems**		**2W2N (H306Y)**	**2W2Q (D374H)**	**2W2O (D374Y)**	**2W2P (D374A)**
AMBER14SB	Δ(ΔE_es_ + ΔG_PB_)	4.1	−1.1	7.3	−8.6
	Δ(ΔE_vdW_ + ΔG_np_)	8.3	4.9	−0.7	4.4
	ΔΔG_PBSA_	12.4	3.8	6.6	−4.2
	Δ(–TΔS_solute_)	−3.1	−4.0	−8.0	−4.2
	ΔΔG_bind_	9.3	−0.2	−1.4	−8.4
EPB	Δ(ΔE_es_ + ΔG_PB_)	−11.0	−14.5	−8.9	−9.7
	Δ(ΔE_vdW_ + ΔG_np_)	10.2	8.1	4.7	5.7
	ΔΔG_PBSA_	−0.8	−6.4	−4.2	−4.0
	Δ(−TΔS_solute_)	−2.9	2.4	−0.5	−2.3
	ΔΔG_bind_	−3.7	−4.0	−4.7	−6.3

*All energy values are in kcal/mol. ΔΔG_PBSA_, Δ(–TΔS_solute_) and ΔΔG_bind_ are the differences of ΔG between WT (2W2M) and those of four mutants*.

## Results and discussion

### Relative binding energy ΔΔG_bind_ calculated from the standard amber force field

The EGFA domain makes contact with the catalytic domain of PCSK9 only. The interaction surface is dominated by an anti-parallel β-sheet between EGFA and the exposed side of a β-hairpin loop of PCSK9ΔC. One important residue of this loop is D(Asn)374 (highlighted in red in Figure 1). Mutation of residue D(Asn)374 to H(His)/Y(Tyr)/A(Ala) leads to GOF mutants D374H/D374Y/D374A, which results from enhanced binding affinity to the interaction with EGFA. MD simulations and binding energy analysis were performed to the WT PCSK9ΔC-EGFA (2W2M) and GOF mutants PCSK9ΔCD374A-EGFA (2W2P), PCSK9ΔCD374Y-EGFA (2W2O), PCSK9ΔCD374H-EGFA (2W2Q) respectively. The detailed binding energy for all five systems was listed in Table S1. To analyze the influence of mutation on binding affinity, the relative binding energy differences compared to the wild type were listed in Table 1 (*T* = 300 K). The binding energy difference ΔΔG_bind_ of the systems GOF mutations (D374H/D374Y/D374A) is −0.23, −1.46, and −8.43 kcal/mol respectively. Compared with the data of WT PCSK9ΔC-EGFA, ΔΔG_bind_ are all negative values which indicate that these three mutants bind with and EGFA stronger than WT. However, in one system, EGFA H306Y mutant, the calculated relative binding free energy is 9.38 kcal/mol which is inconsistent with experimental results. Previous studies using the TR-FRET assay suggest that the EGFA mutant H306Y showed a three-fold enhancement in affinity for PCSK9 (McNutt et al., 2009). According to the MD simulation with standard amber force, the binding affinity of EGFA-H306Y mutant is weaker than the wild type.

### The hydrogen bond analysis of structure from simulation with amber force field and EPB

There are four elementary forces contributing to the protein-protein binding process: hydrogen bonds, electrostatic interactions, hydrophobic effect, and van der Waals interactions. Many theoretical studies on protein-protein interactions indicate that lack of polarization effect in traditional force field may result in incorrect structures in simulation. Effective polarizable bond (EPB) method can provide a good correlation to the traditional force field on the basis of keeping the effective charge character. Previous study (Duan et al., 2010, 2012; Tong et al., 2010) shows that incorporation of polarization effect in MD simulation is important for accurate description of protein dynamics in solvation. We performed MD simulations with EPB force field, which include polarization effects in molecular simulation using effective polarizable charge (Ji et al., 2012; Xiao et al., 2013).

**Figure 2:**
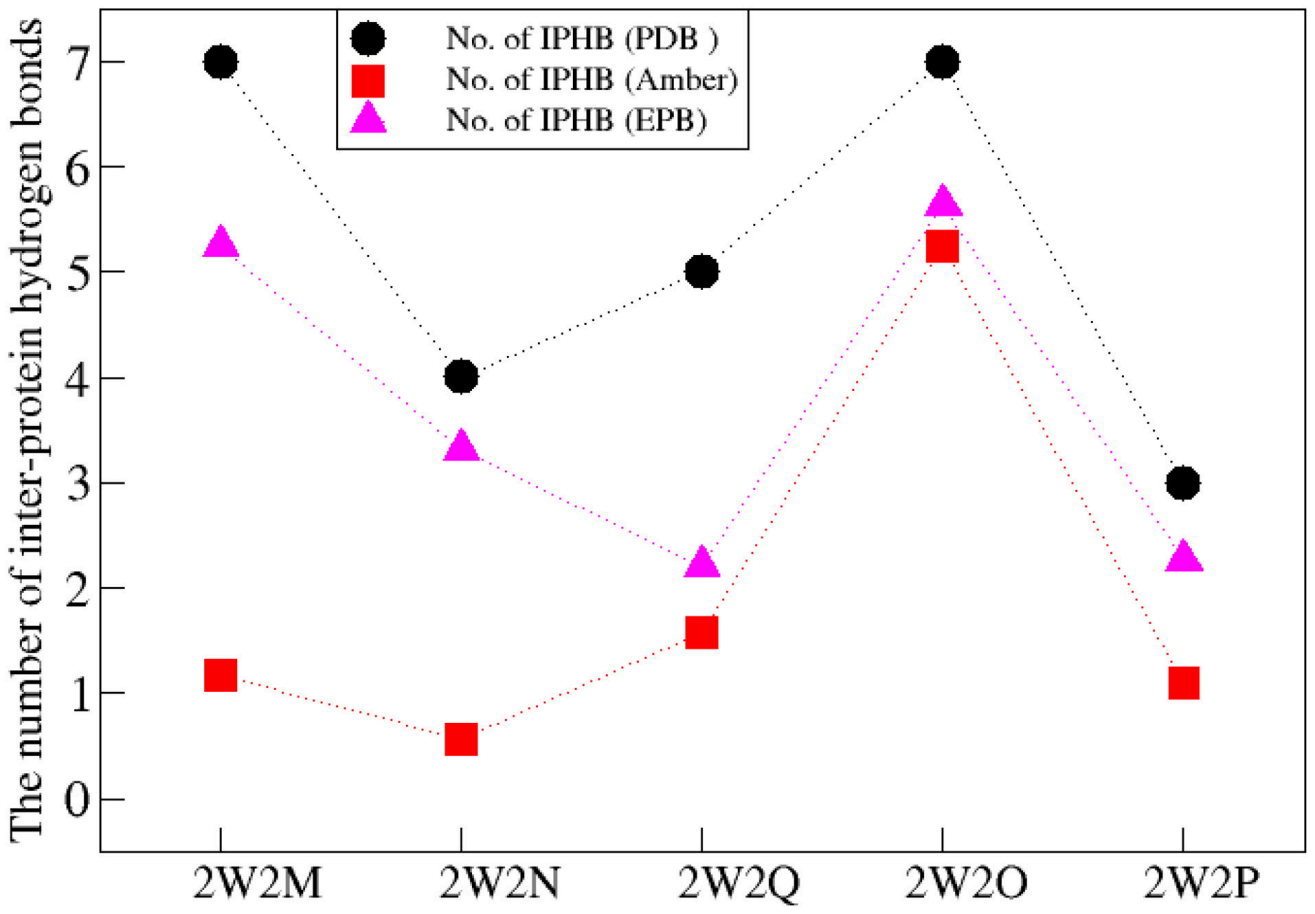
The number of IPHB of five systems obtained from PDB (black circle), MD result using standard amber charge (red square), and EPB (magenta triangle).

The Inter-Protein Hydrogen Bonds (IPHB) in the interaction surface between EGFA and PCSK9 catalytic domain were analyzed and the results were listed in Table 2 (from the trajectory of simulation with EPB) and Table S2 (from the trajectory of simulation with Standard Amber force field). The interaction surface is between an antiparallel β-sheet in EGFA and the exposed side of a β-hairpin loop of PCSK9ΔC. The No. of IPHB in PDB structures and EPB listed in Table 2 was calculated from the initial structures of the PDB files from PDB Bank (2W2P, 2W2O, 2W2Q, 2W2M, and 2W2N) and the trajectories from EPB calculation. These data from crystal structure provide us the information of the hydrogen bonds in the very beginning of MD simulation. Table 2 shows donor, acceptor, and occupancy information for each hydrogen bond. The occupancy was calculated from the MD simulation trajectories by defining that the distance between donor and acceptor is shorter than 4 Å, the angle of donor-acceptor-H-acceptor is larger than 90°. The average number of IPHB of five complexes from MD simulation using PDB, Standard amber charge and simulation EPB was plotted in Figure 2. We can find that the average number of hydrogen bonds in EPB calculations (magenta triangle) was more than those in Standard amber charge calculations (red square) in all the five systems. This plot suggests that more inter protein hydrogen bonds were well-preserved in EPB simulation.

**Table 2:** Hydrogen bond analysis in five protein-protein systems in MD simulations using EPB.

**System**		**Donor**	**Acceptor**	**No. of hydrogen bonds**		**Occupancy (EPB %)**
				**PDB**	**EPB**	
2W2M (WT)	H1 H2 H3 H4 H5	394@O 209@O 207@O 395@OD1 209@O 396@O 207@OG1	209@N 394@N 396@N 207@OG1 387@ND2 207@OG1 395@ND2	7	5.25	100 100 100 96 93 23 13
2W2N (H306Y)	H1 H2 H3	201@OD2 384@OD1 201@OD1 206@O 392@OD1 393@O	389@OH 208@OG 389@OH 384@ND2 204@OG1 204@OG1	4	3.32	99 94 93 40 4 2
2W2Q (D374H)	H1	392@OD1 393@O 205@OG1 392@ND2 207@O 402@O 202@ND1	205@OG1 205@OG1 392@ND2 205@OG1 384@ND2 202@NE2 389@NE2	5	2.21	92 44 40 37 5 2 1
2W2O (D374Y)	H1 H2 H3 H4 H5	390@OD1 389@O 205@O 382@OD1 400@O 391@O 390@ND2 205@OG1 207@O 390@OD1	205@OG1 207@N 391@N 209@N 202@OH 205@OG1 205@OG1 390@ND2 382@ND2 205@N	7	5.64	100 100 100 96 64 32 21 21 17 13
2W2P (D374A)	H1	392@OD1 392@ND2 204@OG1 393@O	204@OG1 204@OG1 392@ND2 204@OG1	3	2.27	100 46 46 35

**Figure 3:**
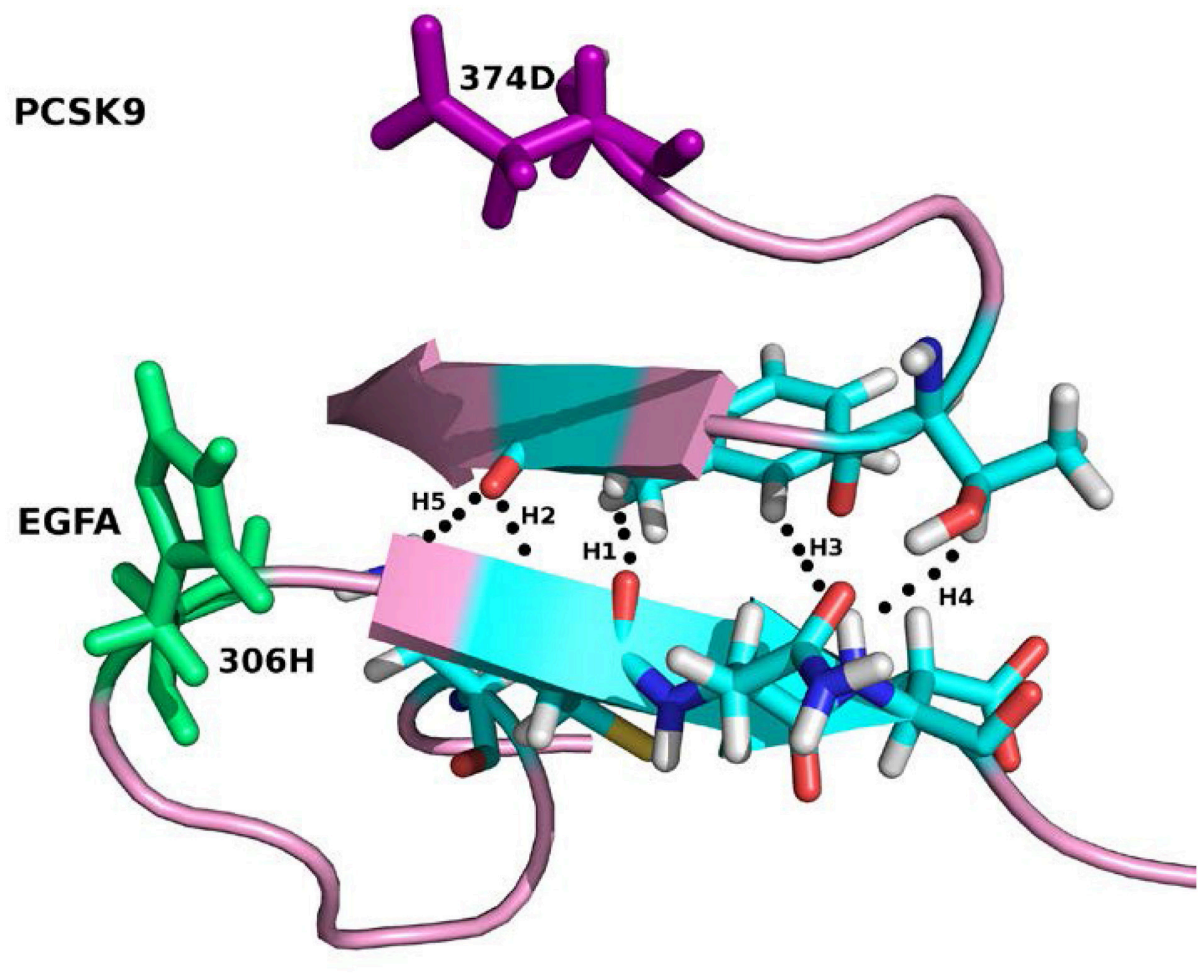
The five strong hydrogen bonds H1, H2, H3, H4, H5 of the complex WT 2W2M. The residues at the mutation site D(Asp)374 and H(His)306 are highlighted in purple and green, respectively.

For the system WT 2W2M, all hydrogen bonds in crystal structure are shown in Figure 3, the residues below belong to EGFA domain and the residues on the above belong to PCSK9 catalytic domain. Figure 3 shows that the 374D(Asn) (purple residue in Figure 3) of PCSK9 had no contact with 306H(His) (green residue in Figure 3) of EGFA domain. There are five strong hydrogen bonds between antiparallel β-sheet. When the 374D(Asn) in WT 2W2M was mutated to 374H(His) or 374Y(Tyr), the structures are very similar to the WT complex. But the side chain of 374H(His)/374Y(Tyr) could form inter-protein hydrogen bonds with EGFA. Thus, for systems 2W2M, 2W2Q, and 2W2O, mutating 374D all lead to additional interaction between PCSK9 and EGFA, resulting in the larger buried surface area at the interface (982 A^2^ for WT 2W2M, 1,045 A^2^ for D374H 2W2Q, 1,139 A^2^ for D374Y 2W2O).

**Figure 4:**
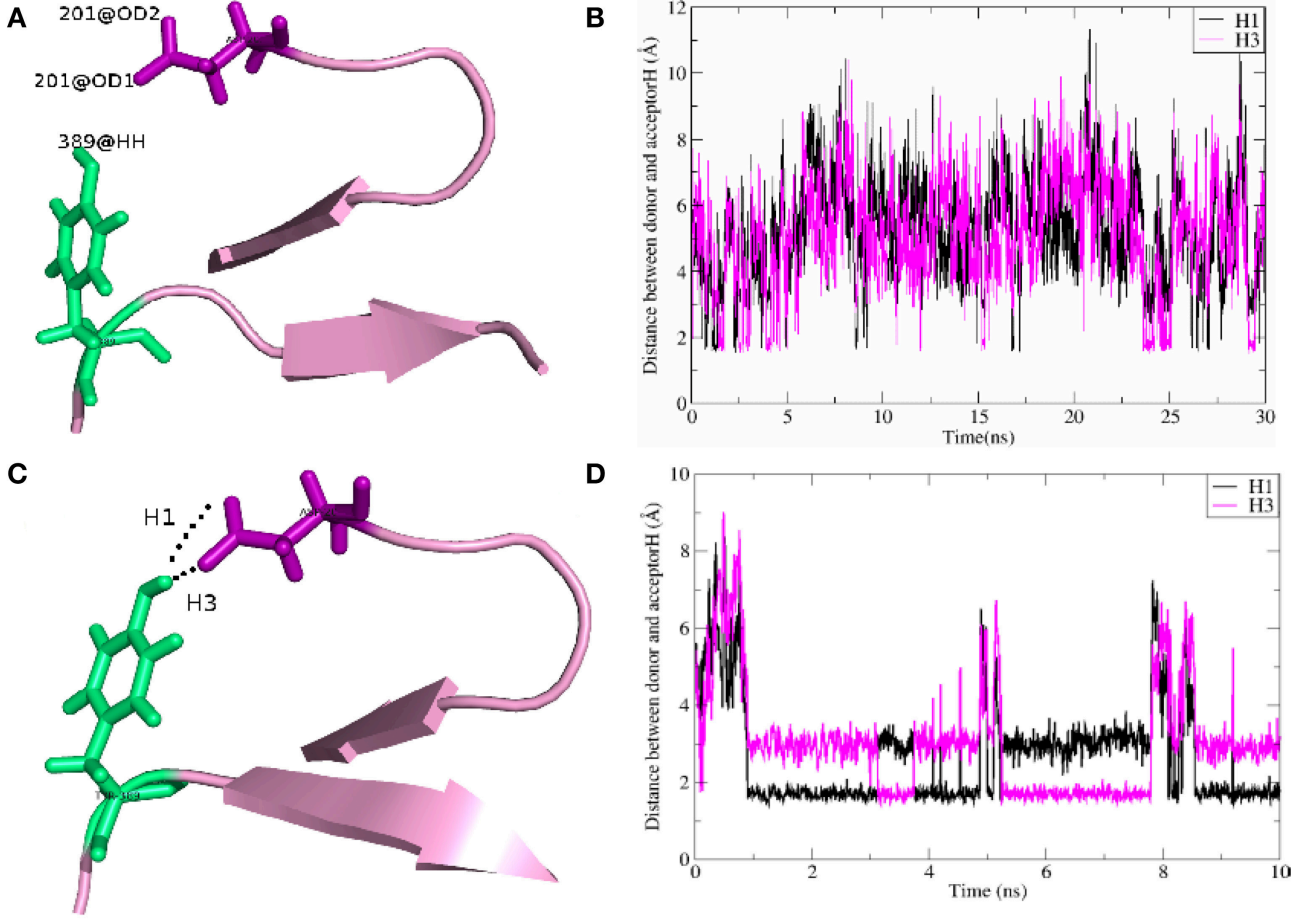
The detail structures of hydrogen bonds H1 and H3 of the complex H306Y (2W2N) and the distance (Å) between donor and acceptor as a function of simulation time. The MD results using standard AMBER charge and EPB charge are shown in figures **(A–D)**, respectively.

As shown in Table 2, for the system WT, there are 7 hydrogen bonds found in initial PDB structure. In the EPB calculation, five strong hydrogen bonds (H1, H2, H3, H4, H5) exist with high occupancy. As to the MD simulation with standard amber charge, only one hydrogen bond exists with occupancy of 100%, and all other hydrogen bonds break in the simulation. The similar results were also found in the mutant complexes 2W2Q and 2W2O. For the system 2W2N (H306Y), the mutation site is on the EGFA domain. There are three strong hydrogen bonds H1, H2 and H3. In the EPB MD simulation, two high occupancy hydrogen bonds (H1 and H3) between D(Asn) residue of PCSK9 and Y(Tyr) residue of EGFA domain still exist. Figure 4 shows the detailed position of H1 and H3. For structure in the MD simulation with standard amber charge, the distances between the donor and acceptor of H1&H3 as a function of simulation time were plotted in Figure 4B. The structure gets from one snapshot of the trajectory were also pictured (Figure 4A). Data in Figure 4 shows that the H1&H3 bonds didn't exist along the simulation time. There is no hydrogen bond interaction between D(Asn) and Y(Tyr) is the simulation using amber charge. However, in the MD simulation with EPB, we can find that the Y(Tyr) side chain forms two hydrogen bonds with the D(Asp) side chain. And the hydrogen bond length indicates that these two hydrogen bonds are really strong bonds (shown in Figures 4C,D).

The structure and hydrogen bond analysis indicate that incorporation of polarization effect in MD simulation is essential for describing protein structure correctly. MD simulation using standard amber force field results in breaking of many important hydrogen bonds and improper predicted binding energy. In simulation with Amber Force field, since many inter protein hydrogen bonds break, the structure sampled from simulation fluctuate a lot.

### Relative binding energy ΔΔG_bind_ calculated from EPB

Since structure sampled from the MD simulations with EPB is more accurate, we did MM/PBSA calculations to compute the binding energy differences ΔΔG_bind_. Similar to the above section, the binding energy differences ΔΔG_bind_ calculated from EPB of the five systems were also listed in Table 1 (*T* = 300 K). Compared with the data calculated by trajectory from simulation with standard amber charge, the data from EPB are more reasonable, especially for the mutate system 2W2N (H306Y), with a negative binding energy ΔΔG_bind_ = −3.73 kcal/mol. The binding energy difference ΔΔG_bind_ follows the order WT < H306Y < D374H < D374Y < D374A. In simulation with Amber force field, many hydrogen bonds break indicates that the structure fluctuate a lot during the simulation. Binding free energy calculated from those largely fluctuated structures may vary a lot during simulation time and randomness may dominate the final value. However, the structure is relatively stable in simulation with EPB force field and the binding free energy calculated from stable structures is more acceptable.

For the system H306Y (2W2N), as we discussed above, when the 306His residue in EGFA domain was mutated to Tyr, two more inter-protein hydrogen bonds form between 306Tyr and 374Asn residue in PCSK9 catalytic domain. In WT 2W2M complex, these two residues do not interact directly. Thus, this mutation (306His residue in EGFA domain mutated to Tyr) leads to enhanced binding between two proteins. Compared with binding energy ΔΔG_bind_ of the complex WT, the data for the H306Y complex is ΔΔG_bind_ = −3.73 kcal/mol.

When the 374D(Asn) residue was mutated to His/Tyr, as mentioned above, the surface buried on PCSK9-EFGA for D374H and D374Y mutants are 1045 and 1139 A^2^, respectively. Compared with the data of WT (982 A^2^), the surface area increases. The buried surface area at the interface increases due to packing of the aromatic side chain of PCSK9 mutate residue His/Tyr against EGFA Leu residue. Thus, the binding energy ΔG_bind_ of the two systems D374H/D374Y increases compared with the data of WT.

**Figure 5:**
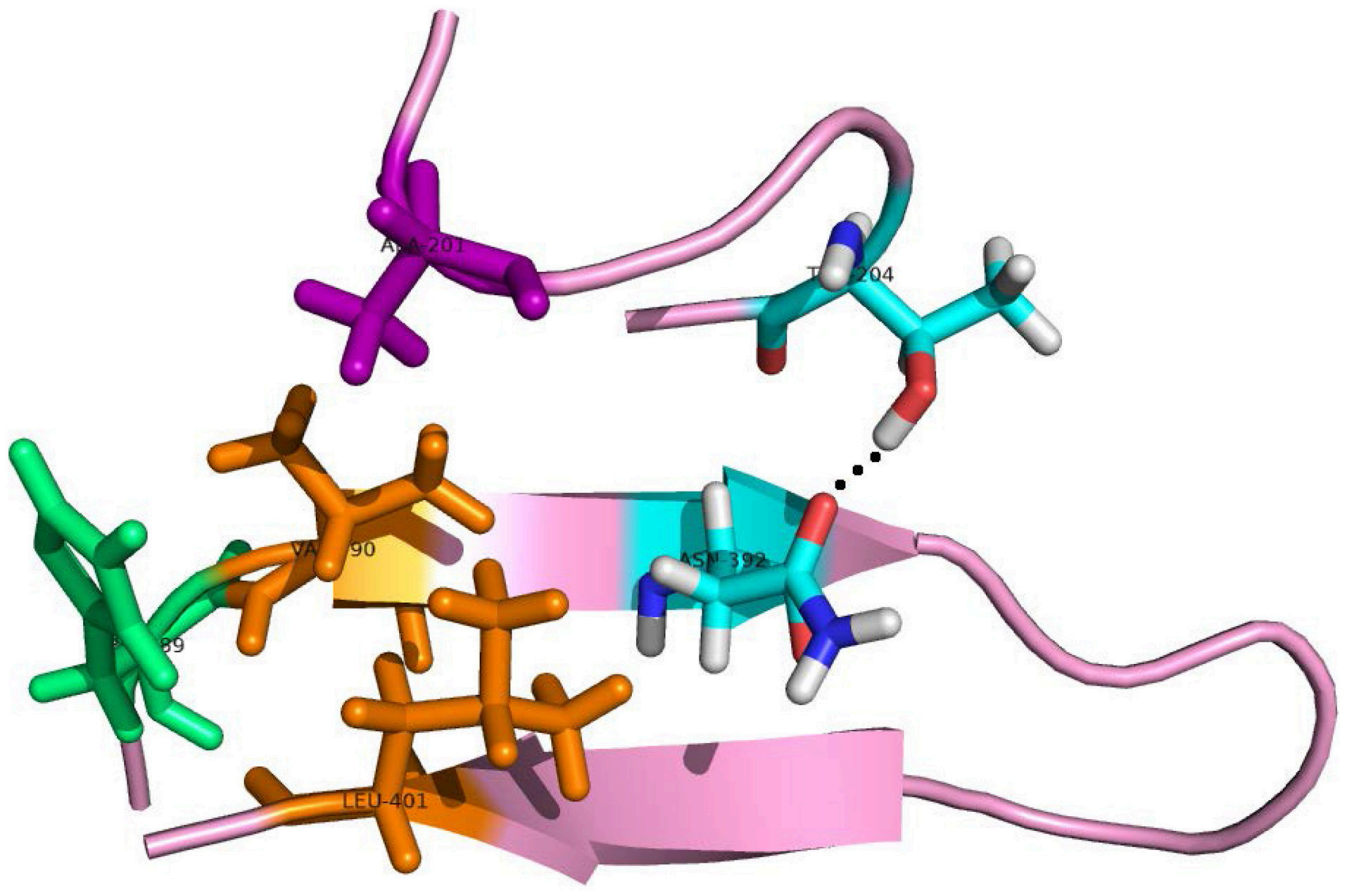
The detailed hydrogen bond structures of the complex D374A (2W2P). The mutated residue ALA (purple) forms a hydrophobic pocket with Val and Leu (orange).

As to the system 2W2P(D374A), the charged D(Asn) residue was replaced by non-polar A(Ala) residue, this A(Ala) residue is very close to the residue L(Leu) and V(Val) which belongs to EGFA domain (Figure 5, the position of L&V were highlighted in orange). The L(Leu) and V(Val) residue have two CH3 each, and these four CH3 forms a hydrophobic pocket with the CH3 in residue A(Ala) on PCSK mutate site. The mutation of D(Asn) into A(Ala) results in an increased affinity for the EGFA because of this hydrophobic packing effect. When the charged residue was replaced by non-polar residue, there is only one strong hydrogen bond formed in the 2W2P system (Table 2), indicating that the electrostatic effect is weak. The order of the hydrophobic parameter is D < H < Y < A, and the ΔΔG_bind_ followed the same trend. Our calculation suggests that if the D(Asn) residue was replaced by hydrophobic amino acid with a larger hydrophobic parameter, we will get a more tightly bound complex.

## Conclusion

To understand the interaction between PCSK9 and LDLR, we performed MD simulations of the wild type (WT) PCSK9ΔC-EGFA complex and the gain-of-function (GOF) mutants PCSK9ΔCD374A-EGFA, PCSK9ΔCD374Y-EGFA, PCSK9ΔCD374H-EGFA, PCSK9ΔC-EGFAH306Y. Result from MD simulation using standard amber charge showed that the predicted relative binding free energy is not consistent with experimental findings, especially for the system H306Y(2W2N). Previous studies (Ji and Zhang, 2012) suggest that many failures in computational predictions are due to incorrect force fields in the simulation. Lack of polarization is one of the most significant defects of many force fields. The standard Amber force fields use fixed partial charge to describe the inter molecule electrostatic interaction, which is not able to include polarization effect in the simulation. Thus, we did MD simulations with EPB, which is an efficient method to include polarization effects in MD simulation. The results show that most of the inter protein hydrogen bonds are well kept in the EPB MD simulation. However, many important hydrogen bonds no longer exist in the simulation with the standard amber force field. Without inclusion of polarization effect in the simulation, the structures sampled from MD simulation may be incorrect and binding affinity calculated from those incorrect structures is not acceptable. MD simulations with EPB charge can get correct structures and get more accurate binding energy ΔG_bind_. When the residue 374D (Asn) was mutated to H(His)/Y(Tyr)/A(Ala), the hydrophobic property of these residues are D(Asn) < H(His) < Y(Tyr) < A(Ala), and we got the same trend of the binding energy difference ΔΔG_bind_ of the systems WT < D374H < D374Y < D374A using simulation with polarization effect included. Our current study suggests that the mutated residue with more hydrophobic property leads to more tightly interaction between PCSK9 and EGFA.

## Author contributions

JC performed MD simulation and organize the result for discussion. LD helped the formulation of the method and coding of the program. CJ developed the EPB force field for MD simulation and participated in the writing and discussion of the paper. JZ directed overall research and participated in the discussion and correction of the paper.

### Conflict of interest statement

The authors declare that the research was conducted in the absence of any commercial or financial relationships that could be construed as a potential conflict of interest.
