# Supplementary material for: Computational Study of PCSK9-EGFA Complex with Effective Polarizable Bond Force Field
Source: Front Mol Biosci. 2018 Jan 15;4:101. doi: 10.3389/fmolb.2017.00101 (PMC5775225; doi:10.3389/fmolb.2017.00101)
Supplement: Supplementary file 1 [file DataSheet1.pdf]

Computational Study of PCSK9-EGFA Complex with Effective Polarizable Bond Force Field

Jian Chen,[‡] Lili Duan,[⊥] Changge Ji,[*,‡,§] and John Zenghui Zhang[*,‡,§]

[‡] *School of Chemistry and Molecular Engineering, East China Normal University, Shanghai 200062, China*

[⊥] *School of Physics and Electronics, Shandong Normal University, Jinan 250014, China*

[§]*NYU-ECNU Center for Computational Chemistry at NYU Shanghai, Shanghai 200062, China*

[*]*Corresponding author: zhzhang@phy.ecnu.edu.cn, chicago.ji@gmail.com*

Table S1. The binding free energies between PCSK9ΔC and EGFA using standard AMBER14SB and EPB force fields, respectively. All energy values are in kcal/mol.

System		2W2M （WT）	2W2N (H306**Y**)	2W2Q (D374**H**)	2W2O (D374**Y**)	2W2P (D374**A**)
AMBER14SB	ΔG_{PBSA}	-55.1±0.7	-42.7±0.7	-51.3±2.2	-48.5±0.9	-59.3±1.1
	$-T\Delta S_{solute}$	36.9±1.8	33.8±1.2	32.9±1.7	28.9±1.5	32.7±1.8
	ΔG_{bind}	-18.2±2.5	-8.9±1.9	-18.4±3.9	-19.6±2.4	-26.6±2.9
EPB	ΔG_{PBSA}	-50.4±0.3	-51.2±1.3	-56.8±0.9	-54.6±1.3	-54.4±0.5
	$-T\Delta S_{solute}$	33.9±2.2	31.0±2.6	36.3±2.6	33.4±2.4	31.6±1.9
	ΔG_{bind}	-16.5±2.5	-20.2±3.9	-20.5±3.5	-21.2±3.7	-22.8±2.4

Table S2. Hydrogen bond analysis for five protein-protein systems using standard AMBER14SB force field.

Systems		Donor	Acceptor-H	Occupancy
**2W2M (WT)**	H1	394@O	209@H	100%
		395@ND2	207@HG1	10%
		391@O	211@HG	4%
		387@ND2	211@HG	4%
**2W2N (H306Y)**		205@OG1	392@HD21	26%
		392@ND2	205@HG1	25%
		384@OD1	209@HG	6%
**2W2Q (D374H)**	H1	392@OD1	205@HG1	92%
		207@O	384@HD21	33%
		393@O	205@HG1	27%
		384@OD1	209@HG	6%
**2W2O (D374Y)**	H2	389@O	207@H	100%
	H3	205@O	391@H	99%
	H1	390@OD1	205@HG1	97%
	H4	382@OD1	209@H	70%
		202@OH	387@HE2	63%
		391@O	205@HG1	38%
		400@O	202@HH	22%
		390@ND2	205@HG1	18%
		205@OG1	390@HD21	18%
**2W2P (D374A)**		384@OD1	208@HG	62%
		379@OE2	377@HG1	26%
		392@ND2	204@HG1	13%
		382@OD1	368@HD22	6%
		379@O	368@HD22	3%
		384@ND2	208@HG	2%
		383@OD1	208@HG	1%

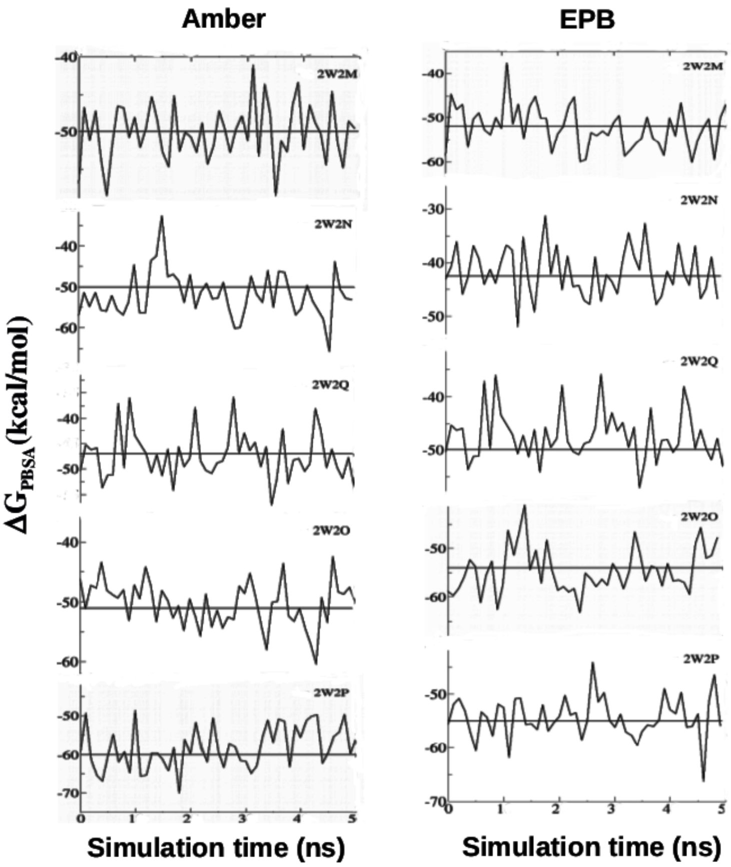

Figure S1. Convergence of the binding free energy ΔG_{PBSA} (gas phase + solvation) during the last 5 ns of simulation from the current trajectories for five systems. The left plot shows the computed result using the standard amber force field and the right plot shows the result using the EPB charge.
